# Supplementary figures and images for: Twelve Months of Time-Restricted Feeding Improves Cognition and Alters Microbiome Composition Independent of Macronutrient Composition
Source: Nutrients. 2022 Sep 24;14(19):3977. doi: 10.3390/nu14193977 (PMC9572159; doi:10.3390/nu14193977)

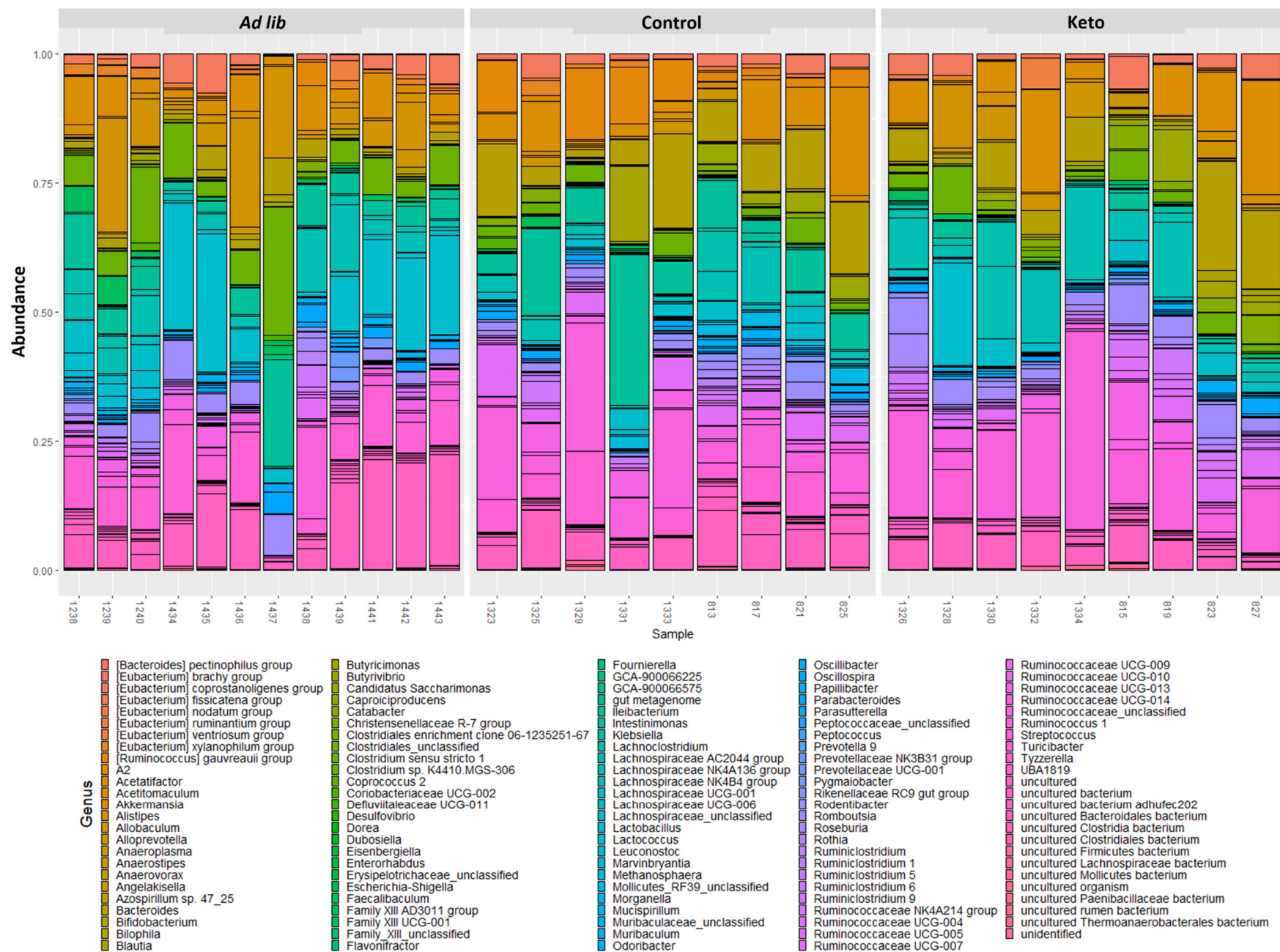

Supplemental Figure S1: Relative abundance at the genus taxonomic level.

Supplement: Supplementary file 1 [file nutrients-14-03977-s001.zip › nutrients-1905069-supplementary.pdf]
